# Quantification of the Link between Timed Up-and-Go Test Subtasks and Contractile Muscle Properties

**DOI:** 10.3390/s21196539

**Published:** 2021-09-30

**Authors:** Andreas Ziegl, Dieter Hayn, Peter Kastner, Ester Fabiani, Boštjan Šimunič, Kerstin Löffler, Lisa Weidinger, Bianca Brix, Nandu Goswami, Schreier Günter

**Affiliations:** 1 AIT Austrian Institute of Technology GmbH, Center for Health and Bioresources, 8020 Graz, Austria; Dieter.Hayn@ait.ac.at (D.H.); Peter.Kastner@ait.ac.at (P.K.); 2 Institute of Neural Engineering, Graz University of Technology, 8010 Graz, Austria; 3 Ludwig Boltzmann Institute for Digital Health and Prevention, 5020 Salzburg, Austria; 4 Alma Mater Europaea ECM, Faculty of Physical Therapy, 2000 Maribor, Slovenia; 2ester.fabiani@gmail.com (E.F.); Bostjan.Simunic@zrs-kp.si (B.Š.); 5 Department of Infectious Diseases, University Medical Centre Ljubljana, 1000 Ljubljana, Slovenia; 6 Institute for Kinesiology Research, Science and Research Centre Koper, 6000 Koper, Slovenia; 7 Albert Schweitzer Institute for Geriatrics and Gerontology, Geriatric Health Care Centers Graz, 8020 Graz, Austria; kerstin.loeffler@stadt.graz.at (K.L.); lisa.weidinger@stadt.graz.at (L.W.); 8 Physiology Division, Otto Loewi Research Center, Medical University of Graz, 8010 Graz, Austria; bianca.brix@medunigraz.at (B.B.); nandu.goswami@medunigraz.at (N.G.)

**Keywords:** Timed Up-and-Go test, tensiomyography, biomedical signal processing, biomedical engineering, biomedical sensors, functional health assessments

## Abstract

Frailty and falls are a major public health problem in older adults. Muscle weakness of the lower and upper extremities are risk factors for any, as well as recurrent falls including injuries and fractures. While the Timed Up-and-Go (TUG) test is often used to identify frail members and fallers, tensiomyography (TMG) can be used as a non-invasive tool to assess the function of skeletal muscles. In a clinical study, we evaluated the correlation between the TMG parameters of the skeletal muscle contraction of 23 elderly participants (22 f, age 86.74 ± 7.88) and distance-based TUG test subtask times. TUG tests were recorded with an ultrasonic-based device. The sit-up and walking phases were significantly correlated to the contraction and delay time of the muscle vastus medialis (*ρ* = 0.55–0.80, *p* < 0.01). In addition, the delay time of the muscles vastus medialis (*ρ* = 0.45, *p* = 0.03) and gastrocnemius medialis (*ρ* = −0.44, *p* = 0.04) correlated to the sit-down phase. The maximal radial displacements of the biceps femoris showed significant correlations with the walk-forward times (*ρ* = −0.47, *p* = 0.021) and back (*ρ* = −0.43, *p* = 0.04). The association of TUG subtasks to muscle contractile parameters, therefore, could be utilized as a measure to improve the monitoring of elderly people’s physical ability in general and during rehabilitation after a fall in particular. TUG test subtask measurements may be used as a proxy to monitor muscle properties in rehabilitation after long hospital stays and injuries or for fall prevention.

## 1. Introduction

Frailty is a syndrome that is associated with a high risk for adverse health outcomes, including mortality, institutionalization, hospitalization, and falls [1]. Falls especially are a major public health problem in older adults. One in three persons over 65 years of age falls at least once a year [2,3]. Age-related loss of muscle mass and function, as well as muscle strength, are markers of the frailty syndrome and, as a consequence, are associated with a high risk of falls [4]. These factors can also be described by the term and disease called sarcopenia, which is a very prevalent phenomenon in aging [5]. Muscle weakness of the lower and upper extremities has been shown to be a risk factor for falls, including injuries and fractures [6]. The muscle mass declines constantly throughout the lifespan. People over 75 years of age especially are losing up to 0.64% of their skeletal muscle mass per year. This substantial loss of muscle mass is accompanied by an even steeper decline in muscle strength, power, and function. Episodes of prolonged muscle disuse further accelerate the degradation of muscle and physical performance [7] and extend the time needed for muscle mass recovery [8]. Immobility during hospital stays is a common example, where people must lie in bed for several days to weeks [9]. Early detection of the loss of muscle mass and strength could improve the prevention, treatment, and sometimes even reversion of muscle atrophy by an effective intervention [10]. Counter measures such as continuous neuromuscular training and mobilization are only effective if initiated in a timely manner [11].

A high level of variability in balance and gait has been reported as a possible cause of falls. An assessment of these factors by methods such as the Timed Up-and-Go (TUG) test can improve the identification of subjects at risk of first-time falling [12,13]. The TUG test is a test of basic mobility maneuvers. The time is taken while a person stands up from a chair, walks three meters, turns, walks back, and sits down [14]. Although this parameter is often used to identify frail subjects and fallers [15,16,17], systematic reviews reported limited ability for the TUG test to predict future falls [18,19]. However, many authors discovered the subtask times for sit-to-stand (walking forward, turning, walking back, and turn-to-sit) to be more meaningful than the total time of the complete TUG test. While Ansai et al. [20,21] used TUG subtasks to distinguish between fallers and non-fallers, as well as non-frail and frail persons, authors such as Li et al. [22] used them in Parkinson’s disease patients. Mirelman et al. [23] found cognitive impairments to be associated with TUG subtasks but not with the overall duration of performance. For this purpose, they used a sensor that measured acceleration and angular velocity. Moreover, Hsieh et al. [24] presented machine learning-based TUG subtask segmentation algorithms to reveal important clinical information from accelerometer, gyroscope, and magnetometer data from subjects with total knee arthroplasty.

Besides gait parameters, lower extremity weakness was reported as a clinically important risk factor for fallers [25]. Tensiomyography (TMG) can be used as a non-invasive tool to assess the functional capabilities of skeletal muscles. It measures the radial muscle belly enlargement after an isometric twitch contraction of a single muscle. The obtained parameters such as contraction time (T_c_), delay time (T_d_), and the maximal displacement amplitude (D_m_) can provide valuable information regarding muscle contraction characteristics [26,27]. A longer T_c_ and T_d_ were found to be associated to a lower proportion of type I muscle fibers. Higher D_m_ was found to be related to decreased muscle stiffness [28,29] and to be correlated to muscle atrophy [30,31].

In elderly people, muscle strength declines more rapidly in the lower extremities than in the upper body. Previous TMG studies have shown that a decreased radial contractile velocity (a ration of D_m_ and T_c_) of the vastus lateralis and biceps femoris was associated with low physical activity and increased age [32].

TUG has been used to evaluate balance and gait, but it has rarely been used to determine the muscle properties. Martinez et al. [33] as well as Filippin et al. [34] used the total TUG time to screen for sarcopenia, achieving moderate accuracy. The relation between TUG results and TMG was also analyzed by previous studies [35].

During the Stand Up-and-Go study, we investigated the correlation between TUG parameters and fall risk. Primary study outcomes were previously published in Ziegl et al. [36]. Initial results from the analysis of the correlation between overall TUG time and TMG parameters have been reported in Fabiani et al. [37]. However, no analysis of the connection between TUG subtasks and TMG parameters has been performed so far. 

In this paper, we present the subtask TUG times based on the measurements of an ultrasonic device and their relation to TMG parameters in four skeletal muscles in elderly subjects, which were harvested during a clinical study in Graz.

## 2. Materials and Methods

### 2.1. Participants

Persons with an age over 65 years from four different nursing homes operated by the Geriatric Health Care Centres Graz were enrolled in the study. The inclusion criteria were mobile, which means able to walk the complete TUG test distance and back (walking aids such as walkers and walking sticks were allowed); living in one of the four participating geriatric nursing homes in Graz; and cognitively competent to give a declaration of consent. Criteria for exclusion were suffering from a tumor or other severe diseases, not living in the above-mentioned nursing homes, and/or being immobile. All participants gave written informed consent prior to inclusion in the study. Ethical approval was obtained by the ethics committee of the Medical University of Graz (GZ:ABT08-182942j2016 PN:8011; 30.1.2018).

### 2.2. Timed Up-and-Go Test Measurements

**Figure 1:**
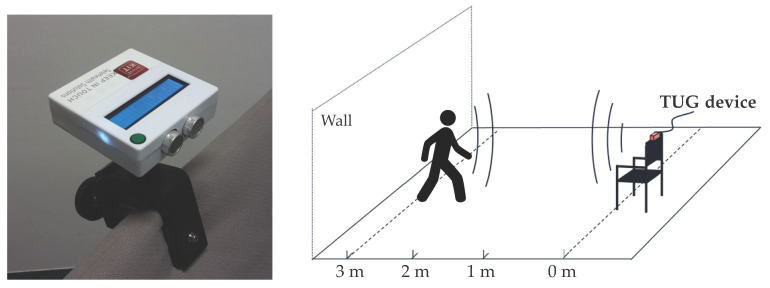
The TUG device is attached to the backrest of a chair (**left**). The measurement setting allowed ultrasonic-based distance measurement. The chair with the attached device was positioned 3.5 m away from a wall. Participants eventually walked 3 m (**right**).

Timed Up-and-Go (TUG) tests were recorded with an ultrasonic-based device attached to the backrest of a chair [38]. The so-called TUG device consisted of an ultrasonic distance sensor, which sends out periodic (10 Hz) high-frequency ultrasonic pulses (40 kHz) within a conical beam pattern. With an accuracy of 3 cm, it was possible to detect participants within a range of 6 m. An LCD display allowed the correct positioning of the chair (3.5 m away from a wall so they eventually walked 3 m). The device posed an acoustic start signal after participants were sitting correctly on the chair (distance <10 cm). The recorded distance over time signals were processed and stored internally on an ARM STM32 microcontroller. These records allowed the calculation of TUG subtasks. Previous investigations identified the following distances from the sensor as appropriate: sit-up/sit-down between 0 and 0.99 m, walk-forward/walk-back between 1 and 3.19 m, and turnaround between 3.20 and 3.50 m [39]. Figure 1 shows the used device, how it was attached to the backrest of a chair, and the measurement setting in front of a wall.

**Figure 2:**
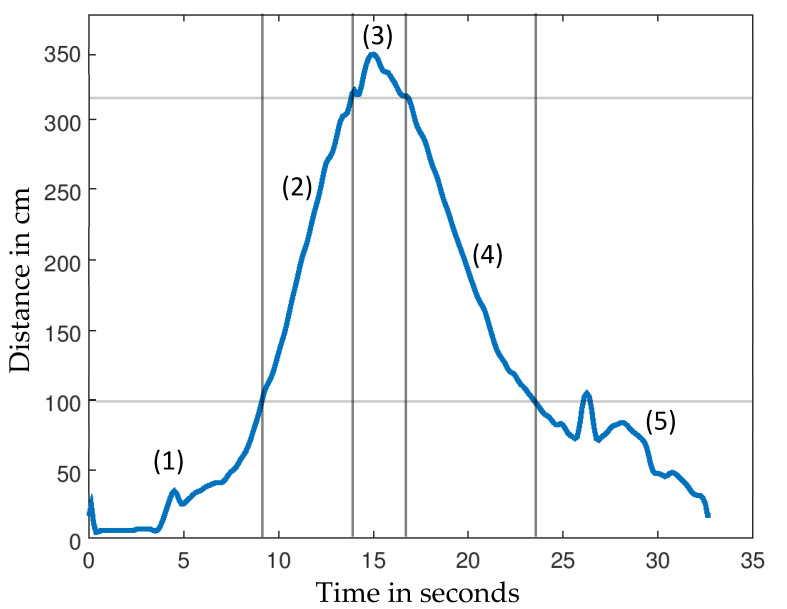
Example signal with the marked subtasks: sit-up (**1**), walk-forward (**2**), turnaround (**3**), walk-back (**4**), sit-down (**5**).

Figure 2 shows a test measurement with the marked subtask times and distances. TUG tests were performed in a controlled setting with a person being present to assist with the procedure. The room was large and empty enough to not detect other obstacles within the beam angle. Two tests were recorded in succession from each participant.

### 2.3. Tensiomyography Measurements

**Figure 3:**
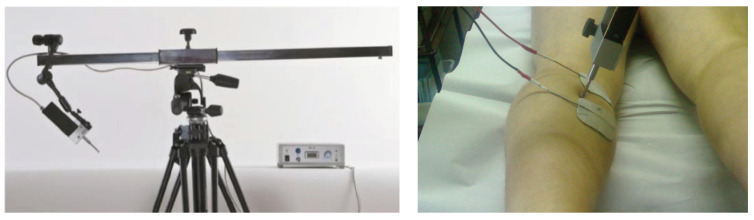
A TMG apparatus with a linear displacement sensor (**left**) was used to detect contractile properties while two self-adhesive electrodes were positioned distal and proximal to the thickest part of the muscle belly (**right**).

**Figure 4:**
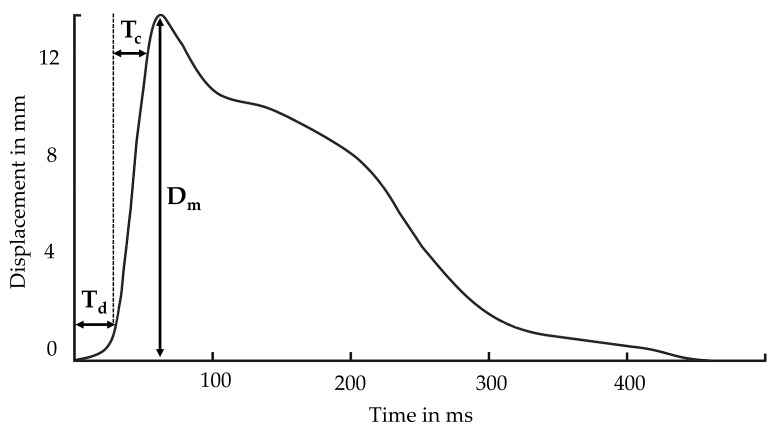
TMG signal with marked parameters: contraction time (**T_c_**), delay time (**T_d_**), and the maximal displacement amplitude (**D_m_**).

TMG was used to detect the contractile properties of four skeletal muscles (biceps femoris BF, gastrocnemius medialis GM, vastus lateralis VL, and vastus medialis VM) from the participants’ dominant leg. A linear displacement sensor assessed radial muscle belly enlargement during maximal isometric twitch contraction (TMG-ZD1, TMG-BMC d.o.o., Slovenia). Participants were asked to relax on a medical bed in supine position (VL, VM) with their knee angle fixed at 30 degrees flexion or prone (GM, BF), with a knee angle fixed at 5 degrees flexion, and ankle in neutral position. Foam pads were used to support the joints. The measuring point for TMG assessment was the thickest part of each muscle belly, as described previously [40]. Briefly, two self-adhesive electrodes (PALS, Axelgaard) were positioned 5 cm distal (cathode) and 5 cm proximal (anode) to the thickest part of the muscle belly (a measuring point identified during voluntary isometric contraction, Figure 3). No skin preparation was carried out. Muscle contraction was triggered with a single maximal rectangular electrical stimulus of 1 ms duration. The linear displacement sensor detected the transverse radial enlargement as a response in time domain. Three contractile parameters were estimated: D_m_ as a maximal amplitude of the response, T_d_ as the time between stimuli, and 10% of D_m_ and Tc as the time between 10% and 90% of D_m_ [26]. Figure 4 shows an example TMG signal with these parameters. In each muscle, two TMG responses were recorded, and an average of estimated parameters was taken for analysis.

### 2.4. Study Design

**Figure 5:**
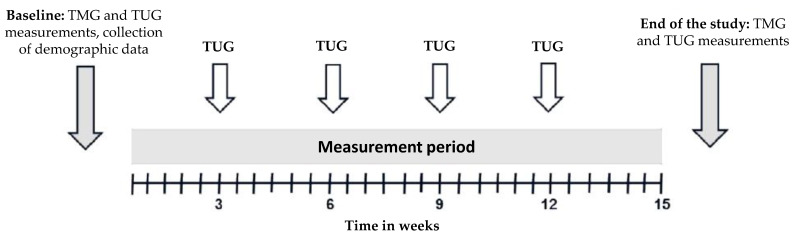
Timeline of the study. Participants could attend six Timed Up-and-Go (**TUG**) test measurements and two tensiomyography (**TMG**) measurements within 15 weeks.

The participants of the study had the chance to attend up to six TUG measurements (one TUG measurement every three weeks). TMG measurements were done at baseline and at the end of the measurement period. Demographic data were collected at baseline. Figure 5 shows the timeline of the complete study.

### 2.5. Statistical Analysis

Values are provided as mean ± standard deviation. Data processing and statistical analysis were done with Python and its libraries numpy, scipy, pandas, and math, as well as the “Predictive Analytics Toolset for Health” (PATH) [41], based on Matlab. The TUG and TMG relations were assessed using the Spearman correlation coefficient, as some TUG parameters were not normally distributed. Correlations with a *p*-value < 0.05 were considered as significant. Additionally, an adjusted significance threshold was calculated by dividing 0.05 with the number of performed correlations between TMG parameters and TUG subtasks (Bonferroni correction). The mean values for all TUG and TMG measurements were calculated for each participant. Scatter plots with regression analysis were created for all significant correlations of the measured TUG and TMG parameters.

## 3. Results

For the analysis, only participants were eligible who attended at least one TUG and one TMG measurement, which was the case for 23 persons (22 female and 1 male). In total, 208 TUG tests and 46 TMG assessments were recorded. The characteristics of the group of participants who performed both TMG and TUG tests are shown in Table 1.

Table 2 shows the mean, minimal, and maximal values of all recorded TMG parameters and TUG test times. The correlation results between TUG test subtask and TMG parameters and the complete TUG test time are shown in Table 3.

**Figure 6:**
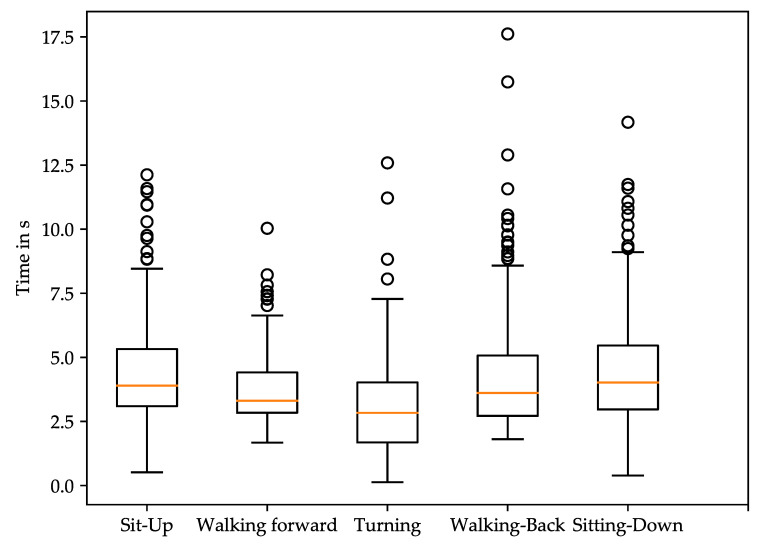
Distribution of Timed Up-and-Go test subtask times for all 23 participants.

The distribution of subtask time values is shown as boxplots in Figure 6. It shows the distribution for all 208 TUG tests that were carried out by the 23 participants.

**Figure 7:**
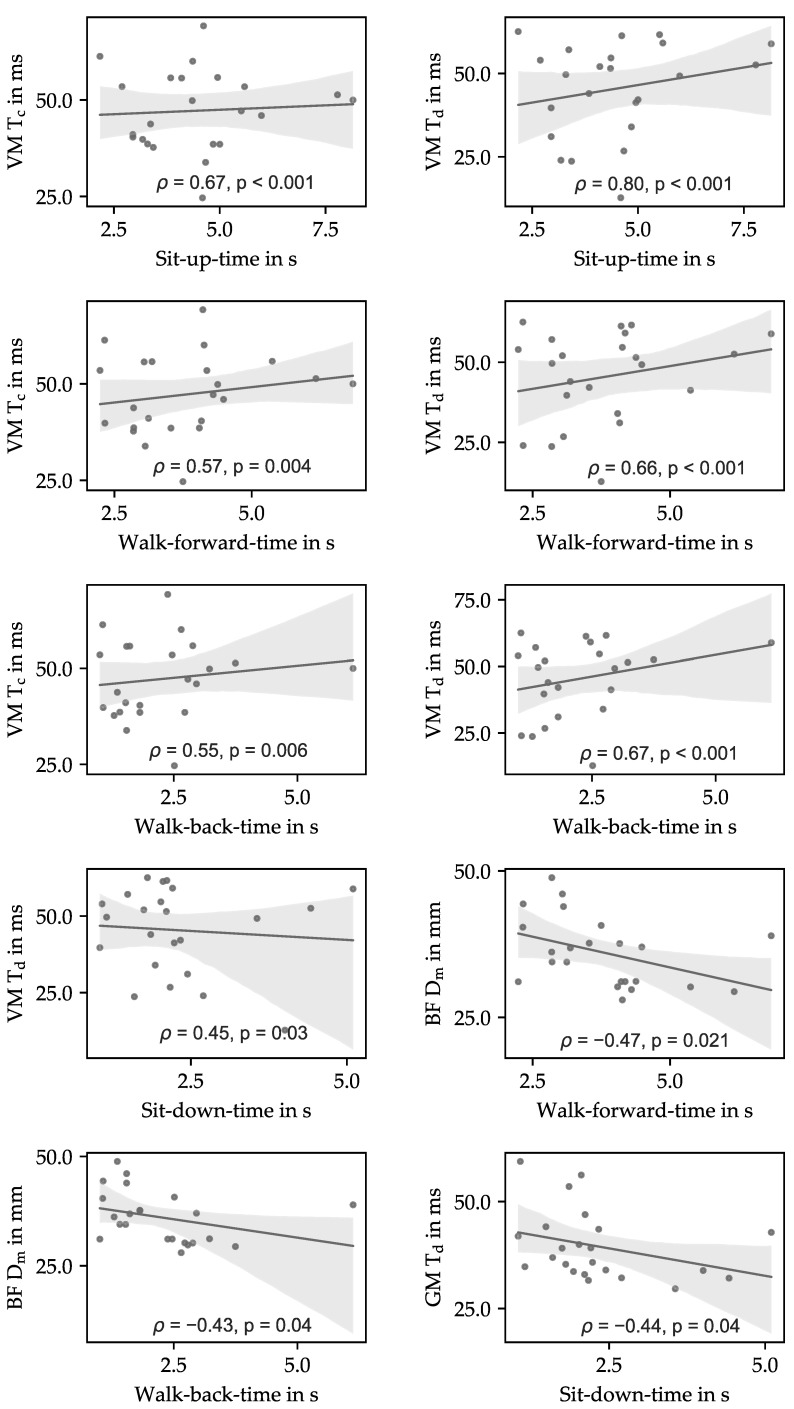
Scatter plots of significant correlations between vastus medialis (**VM**), biceps femoris (**BF**), gastrocnemius medialis (**GM**) tensiomyographic data, and TUG subtask parameters.

Scatter plots of the significant correlations are shown in Figure 7 for the muscles vastus medialis, biceps femoris, and gastrocnemius medialis.

## 4. Discussion

The present results show significant correlations from TUG subtasks times with TMG muscle measurements. Both assessment methods were chosen because they are non-invasive methods that need to be further investigated, if applicable, within elderly people.

TUG measurements with the developed device were well-received by the participants. A staff member was always standing by to operate the device and to convey instructions in order to guarantee reliability of the measurements. Nevertheless, participants had the chance to give themselves a trial without assistance after the measurements. Apart from the received positive feedback on usability, a structured assessment with predefined objectives could help obtain a clearer picture on its acceptance in future investigations.

TMG parameters were determined in four muscle groups in the lower limb: biceps femoris, gastrocnemius medialis, vastus lateralis, and vastus medialis. While the biceps femoris responsibilities are mostly flexion and lateral rotation of the knee, the gastrocnemius medialis muscle additionally supinates the feet. Vastus lateralis and vastus medialis are both responsible for elongating the knee. For this reason, biceps femoris was expected to be mostly active during the walking phases where flexion of the knee is necessary. Vastus lateralis and vastus medialis were expected to be concentrically active during sit-up and walking phase, as well as eccentrically active during sit-down.

This was confirmed for the muscle vastus medialis, as almost all T_c_ and T_d_ values were significantly correlated to all TUG test subtasks, except the turning phase. The relationship between higher T_c_ and T_d_ values and longer TUG test times is comprehensible, as the elongation of the knee is permanently necessary for these subtasks. Previous investigations have shown that higher T_c_ and T_d_ values are correlated to a higher proportion of slow type I fibers [26]. Biceps femoris D_m_ also showed significant correlations but with only the walking phases of the TUG test. This could be due to movements such as flexion and lateral rotation of the knee, which are needed within this task. The fact that this correlation is negative does not come as a surprise since increased connective tissue leads to higher muscle stiffness (lower D_m_), which is a phenomenon that happens especially in elderly people [42,43]. The significant correlations between TMG parameters and TUG test subtasks have shown a symmetric distribution. TMG results of the biceps femoris are only significantly correlated to the walking subtasks, while those of the muscle vastus medialis are also related to the sit-up and sit-down phases. Many TMG parameters of the muscle VM were still significantly correlated to TUG subtasks after applying the adjusted significance threshold according to the Bonferroni correction.

Increased muscle stiffness has a strong, negative effect on the motor performance of the elderly [44]. Counter measures are, e.g., strengthening exercise programs, which were shown to have a decreasing effect on muscle stiffness [43]. Monitoring muscle stiffness could help to make decisions on the success of rehabilitation programs, e.g., after a long bed rest due to a fall, disease, or surgery. If single subtask times can be used to assess certain skeletal muscle properties, a separate evaluation of the complete TUG test and single subtasks would be reasonable. Authors such as Cobo A. et al. [45] presented a solution that just focuses on sit-to-stand and sit-down phases and Fudickar S. et al. [46] an evaluation which neglects the turning phase. This may facilitate TUG device-based statements on both the frailty status (complete TUG test time) and single muscle properties (automated subtask extraction).

The chosen distances for the subtask calculations were based on tests with healthy participants, as well as on field tests with residents of geriatric nursing homes. Nevertheless, the accuracy of these distances could be improved by applying video-based analysis with a larger cohort and comparing them with the distance over time TUG signal.

Subtask times and total TUG times are higher than reported by other authors. Ansai et al. [20] reported mean subtask times of frail persons not higher than 3 s while the values of the participants of this study were all higher. One reason could be a slightly different setting used here, where people had to walk towards a floor mark instead of a wall. Another reason might be that the command to “walk as fast as you feel safe” was not communicated emphatically enough. An advantage of TUG subtask times is that they are easy to measure even without a video-based system. Nevertheless, authors reported gait and balance parameters also to be relevant, which cannot be derived yet from the ultrasonic-based device [47].

TUG test signals were mostly continuous without plateau phases. Consequently, most of the longer TUG tests could be attributed to slower-moving participants. Outliers, which can be seen in Figure 6 and Figure 7, are often caused by a delayed reaction time of the participants for various reasons. Preventing such outliers in future investigations could lead to even more significant results, as they seem to be mostly not associated with muscle activity.

Subjects were mostly females (96%). That is why statements on gender-based performances could not be made. Due to a limited number of participants and recordings, the informative value of these findings is limited and needs to be further investigated. Nevertheless, the symmetric distribution of significant correlations gives a hint that these findings could be reproduced in further studies, which are necessary to verify our findings.

## 5. Conclusions

To our best knowledge, our study is the first to report on the relationship between TMG measurements and TUG subtasks. If these findings can be confirmed in future investigations, automated TUG test subtask measurements may be used as a proxy to monitor muscle properties in rehabilitation after long hospital stays and injuries or for fall prevention.

## Figures and Tables

**Table 1:** Descriptive statistics of the 23 participants (22 females).

	Mean ± Std	Min	Max
Age, years	86.74 ± 7.88	67	99
Body height, cm	160.40 ± 10.10	148.0	188.0
Body mass, kg	64.84 ± 12.97	50.0	96.70
Body mass index, kg/m^2^	25.10 ± 3.60	18.61	34.67

**Table 2:** Values of all recorded tensiomyography parameters and Timed Up-and-Go test times.

	Mean ± Std	Min	Max
Tensiomyography Results			
Biceps femoris T_c_, ms	40.79 ± 8.79	26.46	71.83
Biceps femoris T_d_, ms	30.43 ± 5.09	21.96	44.28
Biceps femoris D_m_, mm	4.43 ± 2.62	0.58	10.79
Gastrocnemius medialis T_c_, ms	31.60 ± 6.67	19.02	47.95
Gastrocnemius medialis T_d_, ms	25.82 ± 3.72	20.02	34.04
Gastrocnemius medialis D_m_, mm	2.49 ± 1.33	0.67	7.47
Vastus lateralis T_c_, ms	29.94 ± 8.07	21.12	68.28
Vastus lateralis T_d_, ms	28.08 ± 3.50	20.79	38.00
Vastus lateralis D_m_, mm	3.74 ± 1.68	0.53	8.36
Vastus Medialis T_c_, ms	47.22 ± 12.16	23.61	70.64
Vastus Medialis T_d_, ms	29.07 ± 3.33	21.44	34.11
Vastus Medialis D_m_, mm	5.84 ± 2.42	1.41	11.20
**Timed Up-and-Go test results**			
Timed up and go time, s	20.30 ± 7.07	9.76	48.67
Sit-up, s	4.51 ± 2.12	0.52	12.12
Sit-down, s	4.59 ± 2.34	0.39	14.17
Turnaround, s	3.00 ± 2.00	0.13	17.61
Walk-forward, s	3.78 ± 1.46	1.67	10.03
Walk-back, s	4.41 ± 2.58	1.81	17.61

T_d_—Delay time; T_c_—Contraction time; D_m_—Maximal displacement amplitude.

**Table 3:** Spearman correlation (*ρ*) results between Timed Up-and-Go test (TUG) subtasks, the complete TUG time, and tensiomyographic (TMG) parameters. Cases with *p*-values less than 0.05 are marked as bold. Those with *p*-values less than 0.0042 (Bonferroni correction) are marked with *.

TUG Time												
	Sit-Up		Walking-Forward		Turning		Walking-Back		Sit-Down		Complete TUG Test	
	*ρ*	*p*	*ρ*	*p*	*ρ*	*p*	*ρ*	*p*	*ρ*	*p*	*ρ*	*p*
BF T_c_	−0.30	0.17	−0.3	0.16	−0.12	0.60	−0.33	0.12	−0.15	0.48	−0.31	0.16
BF T_d_	−0.06	0.80	−0.17	0.43	0.07	0.76	−0.17	0.44	0.05	0.81	−0.00	0.99
BF D_m_	−0.29	0.19	**−0.47**	**0.021**	0.09	0.70	**−0.43**	**0.04**	0.05	0.81	−0.28	0.20
GM T_c_	−0.13	0.55	−0.05	0.81	−0.03	0.89	−0.12	0.58	−0.23	0.29	−0.14	0.54
GM T_d_	−0.14	0.52	−0.15	0.48	−0.06	0.80	−0.22	0.32	**−0.44**	**0.04**	−0.25	0.26
GM D_m_	−0.18	0.4	−0.29	0.18	0.39	0.07	−0.26	0.24	0.03	0.90	−0.13	0.55
VL T_c_	0.00	0.99	0.22	0.31	−0.24	0.28	0.13	0.54	−0.13	0.54	0.02	0.94
VL T_d_	0.17	0.45	0.20	0.35	0.26	0.23	0.15	0.50	−0.14	0.53	0.08	0.72
VL D_m_	−0.31	0.13	0.16	0.46	−0.08	0.71	−0.19	0.38	−0.24	0.27	−0.31	0.14
VM T_c_	**0.67**	<0.001 *	**0.57**	0.004 *	−0.13	0.55	**0.55**	**0.006**	0.33	0.12	**0.54**	**0.008**
VM T_d_	**0.80**	<0.001 *	**0.66**	<0.001 *	−0.04	0.85	**0.67**	<0.001 *	**0.45**	**0.03**	**0.70**	**<0.001**
VM D_m_	−0.28	0.19	−0.27	0.20	0.06	0.78	−0.26	0.23	−0.05	0.83	−0.27	0.21

BF—biceps femoris; GM—gastrocnemius medialis; VL—vastus lateralis; VM—vastus medialis; Td—Delay time; Tc—Contraction time; Dm—Maximal displacement amplitude.
